# Identification of Antimicrobial Resistance Determinants in *Aeromonas veronii* Strain MS-17-88 Recovered From Channel Catfish (*Ictalurus punctatus*)

**DOI:** 10.3389/fcimb.2020.00348

**Published:** 2020-07-17

**Authors:** Hasan C. Tekedar, Mark A. Arick, Chuan-Yu Hsu, Adam Thrash, Jochen Blom, Mark L. Lawrence, Hossam Abdelhamed

**Affiliations:** ^1^College of Veterinary Medicine, Mississippi State University, Mississippi State, MS, United States; ^2^Institute for Genomics, Biocomputing and Biotechnology, Mississippi State University, Mississippi State, MS, United States; ^3^Bioinformatics & Systems Biology, Justus-Liebig-University Giessen, Giessen, Germany

**Keywords:** *Aeromonas veronii*, antibiotic resistant, phage elements, comparative genomics, phylogenetic tree

## Abstract

*Aeromonas veronii* is a Gram-negative species ubiquitous in different aquatic environments and capable of causing a variety of diseases to a broad host range. *Aeromonas* species have the capability to carry and acquire antimicrobial resistance (AMR) elements, and currently multi-drug resistant (MDR) *Aeromonas* isolates are commonly found across the world. *A. veronii* strain MS-17-88 is a MDR strain isolated from catfish in the southeastern United States. The present study was undertaken to uncover the mechanism of resistance in MDR *A. veronii* strain MS-17-88 through the detection of genomic features. To achieve this, genomic DNA was extracted, sequenced, and assembled. The *A. veronii* strain MS-17-88 genome comprised 5,178,226-bp with 58.6% G+C, and it encoded several AMR elements, including *imiS, ampS, mcr-7.1, mcr-3, catB2, catB7, catB1, floR, vat(F), tet(34), tet(35), tet(E), dfrA3*, and *tetR*. The phylogeny and resistance profile of a large collection of *A. veronii* strains, including MS-17-88, were evaluated. Phylogenetic analysis showed a close relationship between MS-17-88 and strain Ae5 isolated from fish in China and ARB3 strain isolated from pond water in Japan, indicating a common ancestor of these strains. Analysis of phage elements revealed 58 intact, 63 incomplete, and 15 questionable phage elements among the 53 *A. veronii* genomes. The average phage element number is 2.56 per genome, and strain MS-17-88 is one of two strains having the maximum number of identified prophage elements (6 elements each). The profile of resistance against various antibiotics across the 53 *A. veronii* genomes revealed the presence of *tet(34), mcr-7.1, mcr-3*, and *dfrA3* in all genomes (100%). By comparison, *sul1* and *sul2* were detected in 7.5% and 1.8% of *A. veronii* genomes. Nearly 77% of strains carried *tet(E)*, and 7.5% of strains carried *floR*. This result suggested a low abundance and prevalence of sulfonamide and florfenicol resistance genes compared with tetracycline resistance among *A. veronii* strains. Overall, the present study provides insights into the resistance patterns among 53 *A. veronii* genomes, which can inform therapeutic options for fish affected by *A. veronii*.

## Introduction

*Aeromonas* species are Gram-negative rods in the family *Aeromonadaceae*. They are among the most common bacteria in aquatic environments and have been isolated from virtually all water source types including freshwater, estuarine environments, drinking waters, wastewaters, and sewage (Janda and Abbott, 2010). The disease caused by *Aeromonas* species affects a broad host range, including freshwater fish, amphibians, reptiles, and birds (Barony et al., 2015). Mesophilic *Aeromonas* species such as *A. hydrophila, A. caviae*, and *A. veronii*, are associated with several kinds of human infections, including gastroenteritis, wound infections, septicemia, and respiratory infections (Figueras, 2005; Igbinosa et al., 2012). *A. veronii* is one member of the genus *Aeromonas*, which is known for causing hemorrhagic septicemia in both wild and farmed fish such as channel catfish (*Ictalurus punctatus*) (Liu et al., 2016; Yang et al., 2017), snakehead (*Ophiocephalus argus*), whitefish (*Coregonus clupeaformis*) (Loch and Faisal, 2010), obscure puffer (*Takifugu obscurus*), Nile tilapia (*Oreochromis niloticus*) (Hassan et al., 2017), and common carp (*Cyprinus carpio*) (Sun et al., 2016). In recent years, an increasing number of *Aeromonas* species have become associated with disease of predominantly freshwater fish in most countries (Goni-Urriza et al., 2000), which results in causing severe outbreaks in different important aquaculture industries.

Sulfonamides potentiated with trimethoprim or ormethoprim, oxytetracycline, florfenicol, and erythromycin are the most commonly used antimicrobial (AM) agents for treatment of *Aeromonas*-related diseases in global aquaculture (Serrano, 2005). Although the judicious use of AM-medicated feeds is important for treatment purposes when faced with outbreaks of bacterial infections (Okocha et al., 2018), a substantial number of reports suggest that indiscriminate use of AMs can foster selection pressure and enable development of multi-drug resistant (MDR) bacteria in aquatic environments (Arslan and Küçüksari, 2015). MDR in bacterial pathogens can result in therapeutic challenges for control of bacterial diseases (Marshall and Levy, 2011).

Over the past two decades, *A. veronii* has gained epidemiological and ecological importance by several research groups due to its potential as an opportunistic and primary pathogen for fish and the prevalence of MDR strains (Sanchez-Cespedes et al., 2008). Previous studies have reported the isolation of MDR *A. veronii* strains from different regions of the world such as Sri Lanka (Jagoda et al., 2017), China (Yang et al., 2017), and United States (Abdelhamed et al., 2019; Tekedar et al., 2019). The resistance elements in *Aeromonas* species are often harbored in mobile genetic elements such as class 1 integrons, plasmids, IS elements, transposons, and genomic islands (Piotrowska and Popowska, 2015). These mobile elements can facilitate the spread of resistance among bacteria via transduction and conjugation (Sanchez-Cespedes et al., 2008; Hossain et al., 2013; Piotrowska and Popowska, 2015). Therefore, it is important to investigate the pattern of resistance, genetic relatedness, and mobile elements in *Aeromonas* species. High-throughput sequencing provides an opportunity to detect MDR bacteria, discover potential resistance mechanisms, and explore the mechanisms underlying resistance gene transfer (Liu et al., 2012).

The purpose of the current work was to uncover mechanisms of resistance in *A. veronii* strain MS-17-88, which was isolated from catfish in the southeastern U.S., and to assess the diversity, resistance profiles, and mobile elements in a large collection of *A. veronii* strains. Here we present the draft genome of *A. veronii* and results from a comparative analysis with 52 publicly available *A. veronii* genomes with a special emphasis on patterns of AMR genes and prophage elements distribution. To the best of our knowledge, no study has been published reporting a core-genome based phylogenetic relationship of sequenced *A. veronii* genomes, AMR profiles, and their mobilomes. Overall, our work provides a basis to understand the AMR profile for *A. veronii* in aquatic environments, which is an important step toward curtailing AMR spread and informing a treatment of disease caused by *A. veronii*.

## Materials and Methods

### Bacterial Strains and Data Source for Comparative Genome Analysis

*A. veronii* strain MS-17-88 was recovered from a diseased channel catfish in 2017 from the Aquatic Diagnostic Laboratory at the College of Veterinary Medicine, Mississippi State University. The isolate was confirmed phenotypically as *A. veronii*. A 20% glycerol stock culture was stored at −80°C. *A. veronii* strain MS-17-88 was cultured in brain heart infusion (BHI) agar or broth (Difco) and incubated at 30°C. Fifty-two *A. veronii* genomes were retrieved from the National Center for Biotechnology Information (NCBI) genomes database on October 9, 2018, including five complete genome sequences and forty-seven draft genome sequences (Table 1).

**Table 1:** The 53 *A. veronii* genomes used in comparative genomic analysis.

**Strain names**	**Country**	**Source**	**Level**	**Size (Mb)**	**GC%**	**Scaffolds**	**Genes**	**Proteins**	**Accession**	**References**
Ae52	Sri-Lanka	Goldfish	Contig	4.56	58.7	80	–	–	BDGY00000000.1	Jagoda et al., 2017
MS-17-88	USA	Catfish	Contig	5.18	58.2	12	4,948	4,651	NZ_RAWX01000001.1	This study
ARB3	Japan	Pond water	Contig	4.54	58.8	63	4,074	3,952	NZ_JRBE00000000.1	Kenzaka et al., 2014
CIP 107763	USA	N/A	Contig	4.43	58.8	64	4,040	3,897	NZ_CDDU00000000.1	N/A
VBF557	India	Human	Contig	4.70	58.4	526	4,460	3,325	LXJN00000000.1	N/A
TTU2014-108ASC	USA	Cattle	Contig	4.53	58.7	58	4,103	3,941	NZ_LKJP00000000.1	Webb et al., 2016
TTU2014-108AME	USA	Cattle	Contig	4.53	58.7	62	4,112	3,938	NZ_LKJN00000000.1	Webb et al., 2016
TTU2014-115AME	USA	Cattle	Scaffold	4.53	58.7	53	4,108	3,943	NZ_LKJR00000000.1	Webb et al., 2016
TTU2014-115ASC	USA	Cattle	Contig	4.53	58.7	52	4,102	3,940	NZ_LKJS00000000.1	Webb et al., 2016
TTU2014-142ASC	USA	Cattle	Contig	4.68	58.6	45	4,242	4,065	NZ_LKKF00000000.1	Webb et al., 2016
TTU2014-130ASC	USA	Cattle	Scaffold	4.68	58.6	49	4,243	4,063	NZ_LKJX00000000.1	Webb et al., 2016
TTU2014-134AME	USA	Cattle	Contig	4.68	58.6	50	4,248	4,063	NZ_LKKA00000000.1	Webb et al., 2016
TTU2014-141AME	USA	Cattle	Scaffold	4.68	58.6	48	4,241	4,066	NZ_LKKD00000000.1	Webb et al., 2016
TTU2014-134ASC	USA	Cattle	Contig	4.68	58.6	59	4,242	4,060	NZ_LKKB00000000.1	Webb et al., 2016
TTU2014-143ASC	USA	Cattle	Contig	4.68	58.6	54	4,246	4,061	NZ_LKKH00000000.1	Webb et al., 2016
TTU2014-125ASC	USA	Cattle	Contig	4.68	58.6	58	4250	4,066	NZ_LKJV00000000.1	Webb et al., 2016
TTU2014-141ASC	USA	Cattle	Contig	4.68	58.6	45	4,240	4,062	NZ_LKKE00000000.1	Webb et al., 2016
TTU2014-113AME	USA	Cattle	Scaffold	4.66	58.6	122	4,239	4,037	NZ_LKJQ00000000.1	Webb et al., 2016
TTU2014-130AME	USA	Cattle	Contig	4.68	58.6	64	4,242	4,064	NZ_LKJW00000000.1	Webb et al., 2016
TTU2014-143AME	USA	Cattle	Contig	4.68	58.6	59	4,248	4,066	NZ_LKKG00000000.1	Webb et al., 2016
TTU2014-131ASC	USA	Cattle	Contig	4.68	58.6	70	4,245	4,055	NZ_LKJY00000000.1	Webb et al., 2016
TTU2014-140ASC	USA	Cattle	Contig	4.68	58.6	81	4,249	4,057	NZ_LKKC00000000.1	Webb et al., 2016
pamvotica	Greece	Sediment	Contig	4.92	58.1	21	4,581	4,317	NZ_MRUI00000000.1	N/A
AER397	USA	Human	Scaffold	4.50	58.8	5	4,014	3,888	NZ_AGWV00000000.1	^*^
B565	China	Pond sediment	Complete	4.55	58.7	1	4,100	3,950	NC_015424	Li et al., 2011
CCM 4359	USA	Human	Contig	4.51	58.9	56	4,170	3,908	NZ_MRZR00000000.1	N/A
CECT 4257	USA	Human	Scaffold	4.52	58.9	52	4,101	3,955	NZ_CDDK00000000.1	Colston et al., 2014
AMC35	USA	Human	Scaffold	4.57	58.5	2	4,064	3,918	NZ_AGWW00000000.1	^*^
AVNIH1	USA	Human	Complete	4.96	58.47	2	4,551	4,321	NZ_CP014774.1	N/A
AVNIH2	USA	Human	Contig	4.52	58.9	50	4,071	3,918	NZ_LRBO00000000.1	N/A
LMG 13067	USA	N/A	Scaffold	4.74	58.4	72	4,265	4,055	NZ_CDBQ00000000.1	N/A
126-14	China	Human	Scaffold	4.37	58.6	146	4,114	3,884	NZ_PPTE00000000.1	N/A
FC951	India	Human	Contig	4.67	58.5	231	4,479	4,066	NZ_PKSR00000000.1	N/A
5.28.6	Greece	Fish	Contig	4.61	58.6	98	4,337	4,107	NZ_NNSE00000000.1	N/A
VCK	Greece	Fish	Contig	4.63	58.6	120	4,366	4,133	NZ_NNSF00000000.1	N/A
NS	Greece	European bass	Contig	4.71	58.5	140	4,503	4,244	NZ_NMUR00000000.1	N/A
PDB	Greece	Fish	Contig	4.72	58.5	141	4,542	4,285	NZ_NMUS00000000.1	N/A
AER39	USA	Human	Scaffold	4.42	58.8	4	3,987	3,832	NZ_AGWT00000000.1	N/A
X12	China	Wuchang bream	Complete	4.77	58.3	1	4,440	4,183	NZ_CP024933	N/A
A29	South Africa	Surface water	Scaffold	4.48	58.8	54	4,142	3,979	NJGB00000000.1	N/A
X11	China	Wuchang bream	Complete	4.28	58.8	1	3,901	3,716	NZ_CP024930	N/A
CCM 7244	Germany	Surface water	Contig	4.42	58.9	74	4,069	3,807	NZ_MRZQ00000000.1	N/A
CECT 4486	USA	Surface water	Scaffold	4.41	58.9	66	4,022	3,831	NZ_CDBU00000000.1	Colston et al., 2014
Hm21	Turkey	Digestive tract	Contig	4.68	58.7	50	4,252	4,116	NZ_ATFB00000000.1	Bomar et al., 2013
CB51	China	Fish	Complete	4.58	58.6	1	4,152	3,623	CP015448	N/A
ZWY-AV1	China	Liver	Contig	4.62	58.6	31	4,317	4,153	NZ_PXYZ00000000.1	N/A
Z2-7	China	N/A	Scaffold	4.41	58.7	48	4,092	3,915	NZ_UETI00000000.1	N/A
ZJ12-3	China	Human	Scaffold	4.70	58.4	124	4,380	4,122	NZ_UETM00000000.1	N/A
ML09-123	USA	Fish	Contig	4.75	58.4	32	4,422	4,204	PPUW01000001	N/A
TH0426	China	Catfish	Complete	4.92	58.3	1	4,528	4,282	NZ_CP012504.1	Kang et al., 2016
XH.VA.1	China	Catfish	Contig	5.36	58.5	62	5,207	4,912	NZ_PZKL00000000.1	N/A
XH.VA.2	China	Catfish	Scaffold	4.91	58.1	48	4,637	4,389	NZ_QQOQ00000000.1	N/A
RU31B	N/A	N/A	Scaffold	4.53	58.7	93	4,203	3,976	NZ_FTMU00000000.1	N/A

* *Human Microbiome U54 initiative, Broad Institute (broadinstitute.org)*.

*N/A, Not available*.

### Antibiotic Resistance Phenotypes of *A. veronii* Strain MS-17-88

The AM susceptibility of *A. veronii* strain MS-17-88 was determined by the Kirby-Bauer disk diffusion method (Bauer et al., 1966). Strain MS-17-88 was streaked on Mueller-Hinton agar plates, and the AM disks were applied on the streaked cultures with a Dispens-O-Disc dispenser. The AM agents tested were florfenicol (30 μg), chloramphenicol (30 μg), tetracycline (30 μg), doxycycline (30 μg), oxytetracycline (30 μg), sulfamethoxazole-trimethoprim (23:75; 1.25 μg), sulfamethoxazole (25 μg), erythromycin (15 μg), gentamicin (10 μg), streptomycin (10 μg), spectinomycin (100 μg), amoxicillin/clavulanic acid (30 μg), ampicillin (30 μg), penicillin (10 μg), ceftriaxone (30 μg), cefpodoxime (10 μg), ceftiofur (30 μg), ciprofloxacin (5 μg), enrofloxacin (15 μg), azithromycin (15 μg), nalidixic acid (30 μg), bacitracin (10 μg), and novobiocin (30 μg). These AM agents were selected based on the World Health Organization's list of the most common classes of antimicrobials (aminoglycosides, tetracyclines, macrolides, beta-lactam, phenicols, quinolones, and sulfonamides) that are regularly used in agriculture and aquaculture and linked to human medicine (Done et al., 2015). After 24 h of incubation at 30°C, the zones of inhibition diameter were measured and compared to the criteria of the National Committee for Clinical Laboratory Standards. The assay was performed in triplicate and repeated as two independent experiments.

### DNA Extraction, Whole-Genome Sequencing, Assembly, and Annotation

Genomic DNA of *A. veronii* strain MS-17-88 was extracted using the DNeasy Blood & Tissue Kit (Qiagen., USA) according to the manufacturer's instructions. Genome sequencing was conducted using HiSeq X Ten (Illumina, San Diego, CA, USA) and MinION (Oxford Nanopore Technologies, Oxford, UK), producing approximately 848.86X and 229.35X genome coverages, respectively. Together, the genome coverage is 1077X. Trimmomatic (Bolger et al., 2014) was used to trim Illumina reads, Nanopore reads were corrected with Canu (version 1.6) (Koren et al., 2017), and contig errors were corrected using Pilon (version 1.21) (Walker et al., 2014). Assembly of the Illumina and Nanopore reads into contigs was done using MaSuRCA (version 3.2.4 (Zimin et al., 2013). Average nucleotide identity (ANI) was calculated based on whole genome sequencing using BLAST alignments (Richter and Rossello-Mora, 2009).

### Phylogenetic Tree

A phylogenetic tree was constructed based on the complete core genome of *A. veronii* strain MS-17-88 and 52 *A. veronii* genomes to evaluate taxonomic positions. All publicly available *A. veronii* genome sequences (52 genomes) were downloaded from NCBI. Gene sets of the core genome were aligned using MUSCLE (Edgar, 2004) and concatenated. Concatenated alignment files were used as an input to compute a Kimura distance matrix, which was followed by using the concatenated files for the Neighbor-Joining algorithm as implemented in PHYLP (Felsenstein, 1989).

### Subsystem Coverages and Genomes Structure Variation

*A. veronii* strain MS-17-88 and 52 *A. veronii* genomes were submitted to Rapid Annotations using Subsystems Technology (RAST) for annotation, subsystem categorization, and comparison purposes (Aziz et al., 2008). The following criteria were used for annotation pipeline: classic RAST for annotation, RAST gene caller for open reading frame (ORF) identification, and Figfam (version release70 with automatic fix errors and fix frameshifts options). *A. veronii* strain MS-17-88 genome was compared against 52 *A. veronii* using BRIG (BLAST Ring Image Generator) (Alikhan et al., 2011).

### Prophages

The presence of prophages in the 53 *A. veronii* genomes was determined using PHASTER (PHAge Search Tool Enhanced Release) (Arndt et al., 2016, 2019). Nucleotide sequences from 53 genomes were concatenated using Sequencher 5.4.5 to serve as an input file prior to submission to the PHASTER server. Results from PHASTER were arranged into three categories: score > 90 was considered intact phage element; a score between 70 and 90 was deemed questionable; and score <70 was considered incomplete phage region (Arndt et al., 2019).

### Comparative Analysis of Putative AMR Elements

The potential AMR genes and related elements for each genome were identified using ResFinder 3.1 (Zankari et al., 2012). ResFinder database was downloaded and used in CLC Workbench version 11.0.1 (CLC Bio) for the BLAST search. The contig files for each genome were concatenated, and concatenated nucleotide files were uploaded to CLC Workbench. A BLAST search was run with the following settings: 40% minimum identity and 40% minimum matching length.

## Results and Discussion

### General Genome Features of *A. veronii* Strain MS-17-88

The present study reported the draft genome of *A. veronii* strain MS-17-88 isolated from diseased catfish in the southeastern U.S. The draft genome of *A. veronii* strain MS-17-88 consisted of 5,178,226 bp with 58.6% G+C content and encoded 4,944 predicted coding sequences (CDSs). A total of 181 RNA genes were predicted in the genome including 139 tRNAs, 4 ncRNAs, and 38 rRNAs (12, 13, 13 for 5, 16, and 23 s, respectively). The final assembly contained 13 contigs. The largest contig assembled was 1,457,362-bp length, and the smallest contig was 7,082-bp. The genome has been deposited in GenBank (accession number NZ_RAWX01000000). *A. veronii* contains two biovars (*A. veronii* biovar veronii and *A. veronii* biovar sobria) (Janda and Abbott, 2010). ANI and phylogenetic tree calculation confirmed that strain MS-17-88 belongs to *A. veronii* biovar veronii (ANI score higher than 95%).

### Genotypic and Phenotypic Characterization of *A. veronii* Strain MS-17-88

The Rasfinder and CARD analysis revealed 14 resistance elements in the *A. veronii* strain MS-17-88 genome (Table 2), including beta-lactamase resistance genes (*imiS* and *ampS*), chloramphenicol and florfenicol resistance gene (*floR*), macrolide resistance genes (*mcr-3* and *mcr-7.1*), streptogramin B resistance *vat(F)*, tetracycline resistance genes [*tet(34), tet(35)*, and *tet(E)*], and acetyltransferase genes conferring resistance to phenicol compounds. The disk diffusion results (Table 3) demonstrated that *A. veronii* strain MS-17-88 strain is resistant to phenicol class (florfenicol and chloramphenicol), tetracyclines (tetracycline, doxycycline, and oxytetracycline), macrolides (erythromycin, azithromycin,), aminoglycoside (gentamicin), sulfamethoxazole, beta-lactam class (amoxicillin/clavulanic acid, ampicillin, and penicillin), spectinomycin, bacitracin, and novobiocin.

**Table 2:** Predicted antibiotic resistance genes in *A. veronii* strain MS-17-88.

**Protein name**	**Protein ID**	**Gene**	**% Identity**	**Query/HSP Length**	**Predicted phenotype**
CphA family subclass B2 metallo-beta-lactamase	RKJ87494.1	*imiS*	89.7	767/768	Beta-lactam resistance
Class D beta-lactamase	RKJ86357.1	*ampS*	93.84	795/795	Beta-lactam resistance
Phosphoethanolamine-lipid A transferase	RKJ90059.1	*mcr-3*	67.19	756/1,626	Colistin resistance
Phosphoethanolamine–lipid A transferase	RKJ90059.1	*mcr-7.1*	73.2	1,601/1,620	Colistin resistance
Antibiotic acetyltransferase	D6R50_13775	*catB2*	65.85	201/633	Phenicol resistance
Antibiotic acetyltransferase	D6R50_13775	*catB7*	68.78	235/639	Phenicol resistance
Antibiotic acetyltransferase	RKJ86442.1	*catB7*	66.61	565/639	Phenicol resistance
Vat family streptogramin A O-acetyltransferase	RKJ85500.1	*catB1*	70.79	265/630	Phenicol resistance
Chloramphenicol/florfenicol efflux MFS	RKJ86396.1	*floR*	98.19	1,214/1,215	Phenicol resistance
Vat family streptogramin A O-acetyltransferase	RKJ85500.1	*vat(F)*	69.52	581/666	Streptogramin B resistance
Xanthine phosphoribosyltransferase	RKJ89311.1	*tet(34)*	66.95	340/465	Tetracycline resistance
Na+/H+ antiporter NhaC family protein	RKJ91399.1	*tet(35)*	70.13	231/1,110	Tetracycline resistance
Tetracycline efflux MFS transporter Tet(E)	RKJ91234.1	*tet(E)*	99.92	1218/1,218	Tetracycline resistance
Type 3 dihydrofolate reductase	RKJ87621.1	*dfrA3*	68.93	348/489	Trimethoprim resistance

**Table 3:** Antimicrobial resistance phenotype of *A. veronii* strain MS-17-88.

**Antimicrobial agents**	**Disk content (μg)**	**Diameter of inhibition zone (mm)**	**Sensitivity**
Florfenicol FFC30	30	0	R
Chloramphenicol C30	30	0	R
Tetracycline TE30	30	6.16 ± 0.44	R
Doxycycline D30	30	10.8 ± 0.41	R
Oxytetracycline T30	30	0	R
Sulfamethoxazole	25	0	R
Sulphamethoxazole trimethoprim SXT	25	17.9 ± 0.20	S
Erythromycin E15	15	11.20 ± 0.11	R
Gentamicin GM10	10	16.24 ± 0.40	R
Streptomycin S10	10	11.4 ± 0.23	S
Spectinomycin SPT100	100	12.7 ± 0.14	R
Amoxicillin/clavulanic acid AMC30	30	9.2 ± 0.11	R
Ampicillin AM10	10	0	R
Penicillin P10	10	0	R
Ceftriaxone CRO30	30	29.43 ± 0.31	S
Cefpodoxime CPD10	10	19.63 ± 0.18	S
Ceftiofur XNL30	30	19.76 ± 0.12	S
Ciprofloxacin CIP5	25	24.76 ± 0.14	S
Enrofloxacin E15	15	24.76 ± 0.14	S
Azithromycin AZM15	15	16.3 ± 0.17	R
Nalidixic acid NA30	30	24.8 ± 0.10	S
Bacitracin B10	10	0	R
Novobiocin NB30	30	9.3 ± 0.15	R

*R, resistant; S, sensitive*.

*Data represented as means diameter of inhibition zones (mm) ± SD of two independent experiments in triplicates*.

Resistance to β-lactam antibiotics in *Aeromonas* species is primarily mediated by β-lactamases, whose mode of action involves hydrolyzing the β-lactam ring. Many different β-lactamases have been detected in *Aeromonas* species, such as TEM-, SHV-, OXA-, CMY-, and CTX-M-type β-lactamases. In the present study, *ampS* and *imiS* were detected in *A. veronii* stain 17-88. AmpS is a class 2d penicillinase and ImiS is a class 3 metallo-β-lactamase. The *imiS* gene has been detected in clinical isolates of *A. veronii* biovar sobria (Wu et al., 2012). MS-17-88 also harbors *floR*, which encodes a major facilitator superfamily efflux pump that exports florfenicol (Schwarz et al., 2004). Florfenicol is one among the three approved AMs for use in catfish aquaculture in the U.S (Bowker et al., 2010). Most fish pathogenic bacteria mediate florfenicol resistance through FloR (Dang et al., 2007; Gordon et al., 2008). Dissemination of florfenicol resistance among bacterial pathogens isolated from aquaculture can limit the efficacy of this agent as an important treatment option.

### Phylogenetic Tree

The phylogenetic relationship between the *A. veronii* MS-17-88 genome and 52 other *A. veronii* genomes was assessed. The strains used in this analysis are from different countries and hosts, and they had distinct resistance profiles (Table 1). The phylogenetic tree for the 53 *A. veronii* genomes was built from a core genome of 2,563 genes per genome (135,839 genes in total). The core has 2,538,377 bp per genome (134,533,981 bp in total).

The phylogenetic tree showed that there are multiple highly conserved branches that are separated from the other *A. veronii* genomes. MDR *A. veronii* strain MS-17-88 from U.S. channel catfish, goldfish (*Carassius auratus*) MDR strain Ae5 from China, and *A. veronii* ARB3 from pond water in Japan were clustered together, which may indicate a common origin. Similarly, U.S. channel catfish isolate ML09-123 and China catfish isolate TH0426 were closely related, which also may suggest derivation from the same monophyletic origin despite their geographic disparity. Moreover, dairy cattle isolates and Greece surface sediment isolates (strain pamvotica) formed another closely related group. U.S. human isolates (strains AER 397, CECT 4257, and CCM 4359) and China pond sediment isolate B565 formed another clade. Lastly, U.S. surface water and Germany surface water isolates were closely related, and Greece fish isolates (strains 5.28.6, VCK, NS, and PDB) were clustered together. These data clearly suggest that the ecological niche is a more important factor contributing to relatedness among *A. veronii* isolates than geographical location. However, two isolates (CIP 107763 and VBF557) showed a distinct genetic relationship to the other isolates.

**Figure 1:**
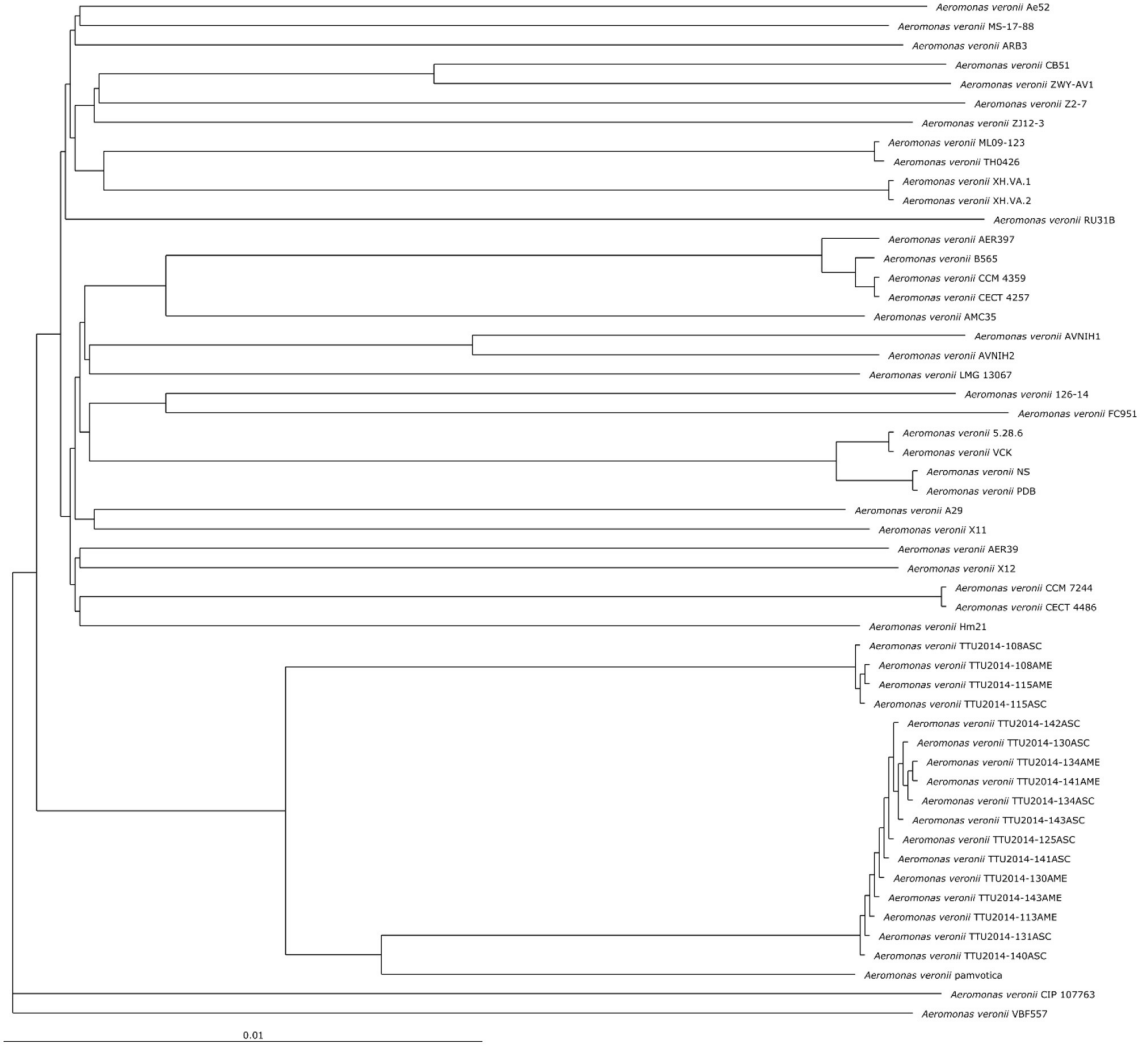
Phylogenetic tree analysis based on the core genomes of *A. veronii*.

It has been postulated that a fish pathogenic *Aeromonas hydrophila* clonal group was transferred to the U.S. channel catfish aquaculture industry from China (Hossain et al., 2014). In the current study, we observed two different clonal groups of *A. veronii* that contain isolates from both U.S. and China. One clade has MDR *A. veronii* strain MS-17-88 from U.S. catfish aquaculture and MDR isolate Ae5 from goldfish in China, and another clade has U.S. channel catfish isolate ML09-123 and China catfish strain TH0426 (Figure 1).

### Subsystems Coverage

**Figure 2:**
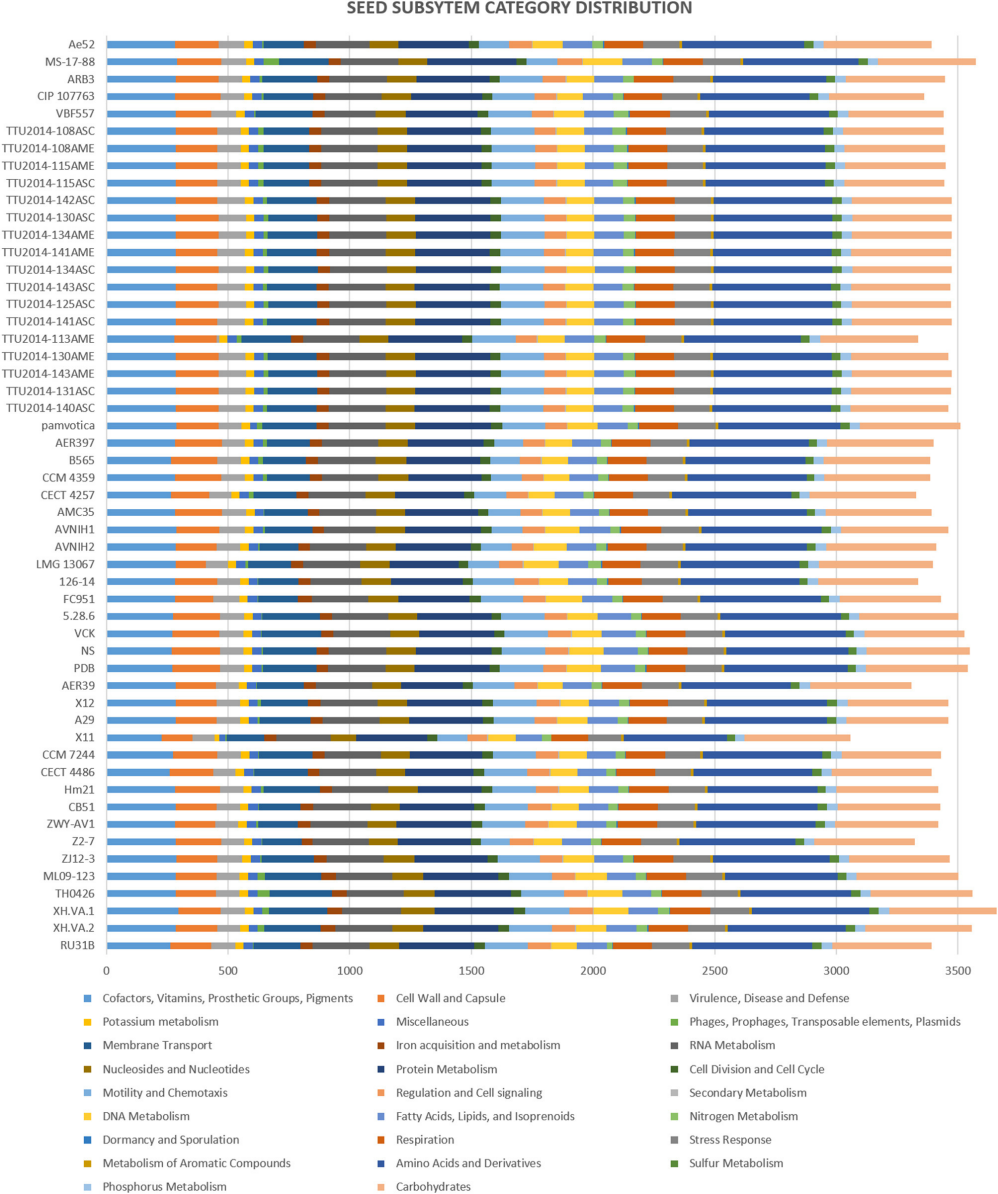
Comparison of functional categories in 53 *A. veronii* genomes based on SEED. Functional categorization is based on roles of annotated and assigned genes. Each colored bar represents the number of genes assigned to each category.

The subsystems categorization based on RAST annotation is shown in Figure 2. SEED subsystem categorization analysis predicted 26 different categories for the evaluated *A. veronii* genomes. The most abundant systems are “amino acid and derivatives,” followed by “carbohydrates” and “protein metabolism.” These subsystems are essential for bacteria to perform basic cellular processes and may indicate the potential ability of *A. veronii* to utilize different kinds of sugars and amino acids available in the environment (Liang et al., 2019). On the other hand, *A. veronii* genomes show remarkably low numbers of mobile genetic elements including phages, prophages, transposable elements, and plasmids. These mobile elements can mediate alteration of genotypes, and these findings may suggest that horizontal gene exchange may not contribute to *A. veronii* genomic variation as much as other species. Interestingly, U.S. catfish isolate strain MS-17-88 carries the most abundant subsystems (63 elements) associated with phages, prophages, transposable elements, and plasmids. Chinese catfish isolate strain TH0426 has the second largest number (52 elements) of phage and transposable elements. Therefore, these two strains (MS-17-88 and TH0426) may have acquired significant genome structure changes by gene acquisitions from mobile elements. It is notable that both strains were isolated from aquatic environments, and we speculate that the aquatic environment may be favorable for genetic exchange and horizontal gene acquisition.

### Genome Structure Variation

**Figure 3:**
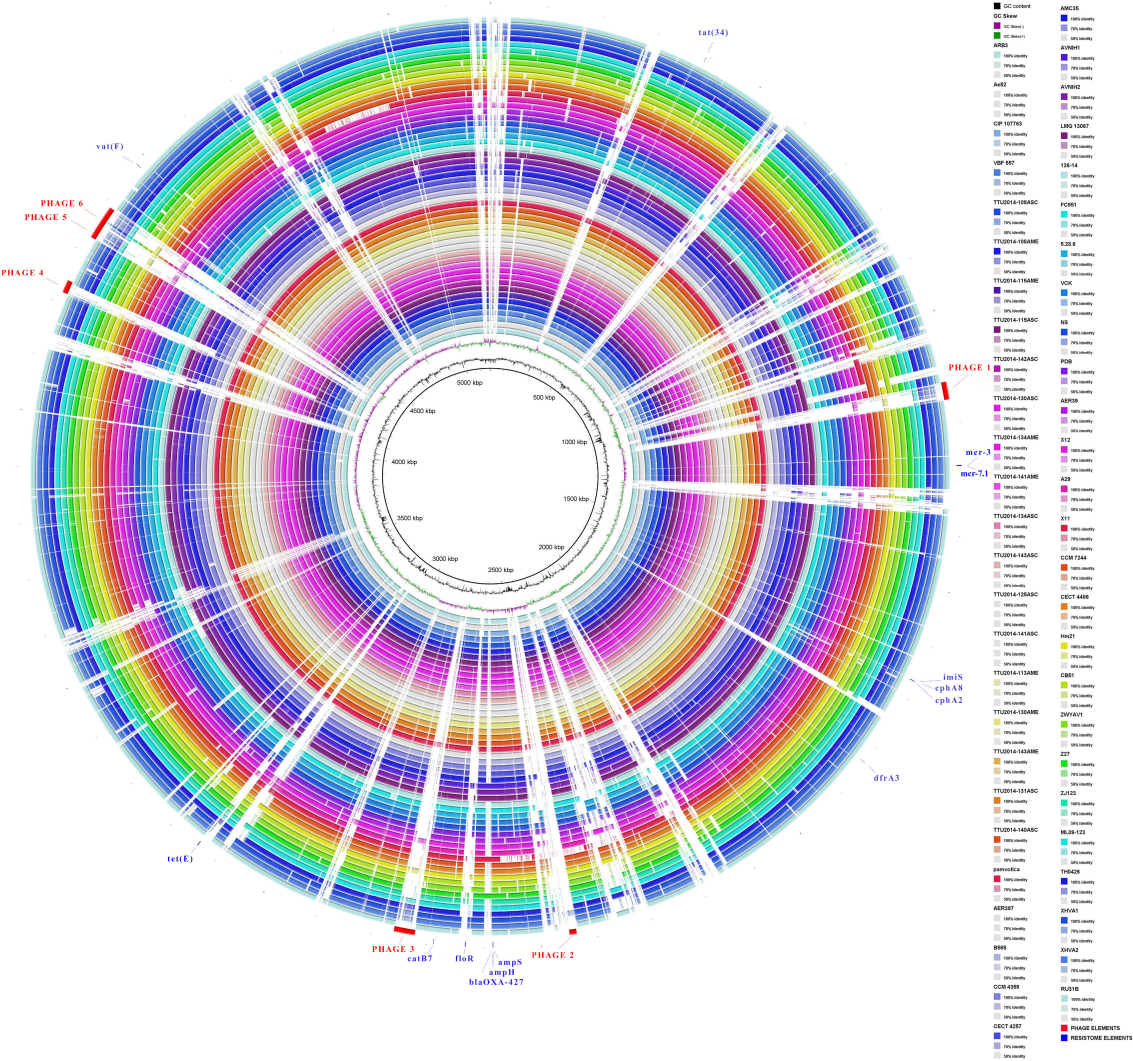
Comparative circular map of the *A. veronii* MS-17-88 genome. Phage regions are highlighted with red color: phage region-1 encodes 41 proteins (31.8 Kb), phage region-2 encodes 12 proteins (12.6 Kb), phage region-3 encodes 39 proteins (37.7 Kb), phage region-4 encodes 31 proteins (22.9 Kb), phage region-5 encodes 45 proteins (48.6 Kb), and phage region-6 encodes 30 proteins (24.1 Kb).

Visualization of the alignment between *A. veronii* MS-17-88 and 52 other *A. veronii* genomes revealed that phage elements, transposons, and genomic islands comprise many of the MS-17-88-specific regions (Figure 3). This suggests that mobile elements, especially phages and transposons, play an important role in *A. veronii* genome variation. These mobile elements can result in genomic rearrangements and evolution through acquisition of novel virulence or antibiotic resistance genes which may result in emergence of new phenotypes (Brown-Jaque et al., 2015).

### Prophages

Bacteriophages are responsible for loci rearrangements and deletions and are recognized as an important element in bacterial evolution (Tinsley et al., 2006). The vast majority of aquatic bacteria (about 70%) are infected with prophages (Chen et al., 2006). Of particular interest, bacteriophages can mediate horizontal gene transfer, including genes encoding virulence factors and antibiotic resistance (Colomer-Lluch et al., 2011). A higher number of phages may represent a concern because they can expand the pathogenicity of a bacterial strain or convert an avirulent strain into a virulent one (Canchaya et al., 2004). Even though phage elements may not be the main spreading factors of resistance elements, recent studies indicate that they can infrequently contribute to the dissemination of these elements (Allen et al., 2011; Enault et al., 2017). In *Aeromonas*, the transfer of resistance gene by phage elements has never been observed previously (Piotrowska and Popowska, 2015).

**Figure 4:**
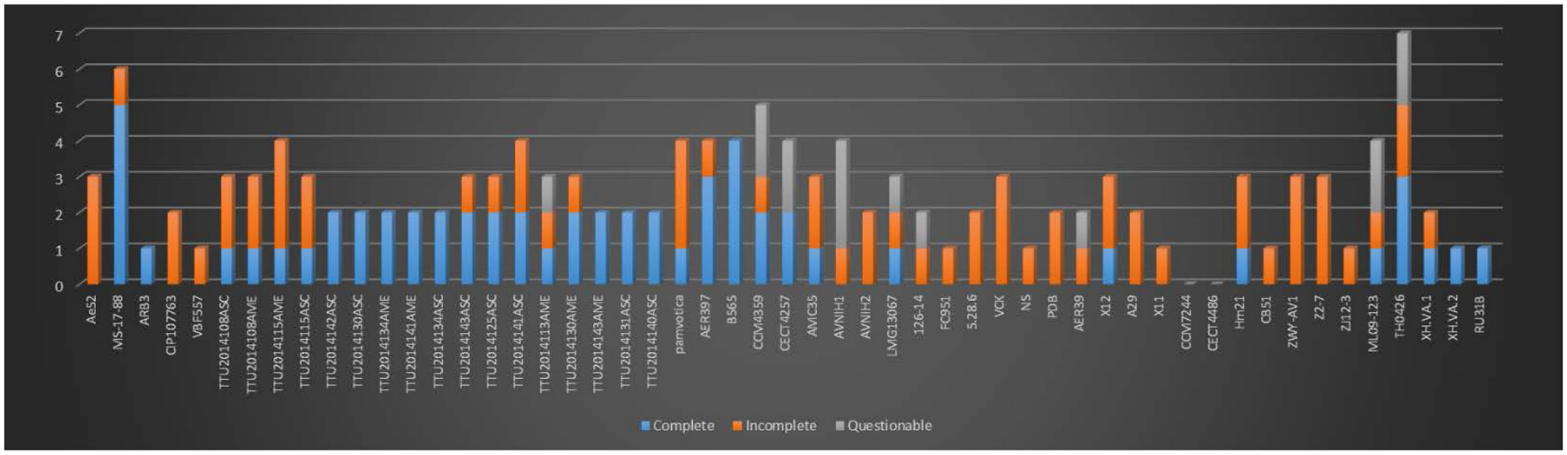
Number of prophages with their completeness profiles in *A. veronii* genomes. Strains FC91, CCM7244, and CECT4486 did not have any prophage elements.

In the present study, we identified 58 intact, 63 incomplete, and 15 questionable phage elements among the 53 *A. veronii* genomes (Figure 4). The average phage element number is 2.56 per genome (136 identified phage elements/53 *A. veronii* strains). In a previous study, *A. hydrophila* genomes were found to harbor an average of 2.91 phage elements per genome (143 identified phage elements/49 *A. hydrophila* strains) (Awan et al., 2018). Almost all the strains carry one or more prophage elements, with the exceptions being strains FC951, CECT4486, and CCM7244, which do not carry any type of prophage. Strains MS-17-88 and TH0426 had the maximum number of identified prophage elements (6 elements per strain). The maximum number of complete prophage elements (5 complete phages) was present in strain MS-17-88 along with one incomplete phage element (Figure 4). The maximum number of incomplete prophage elements (4 incomplete phages) was present in strains TTU2014-108ASC and TTU2014-115AME. Strain MS-17-88 carries three different types of phage elements: vB_AbaM_ME3, phi018P, and RSA1. Interestingly, two of these phage elements (vB_AbaM_ME3 and RSA1) are not carried by any other evaluated *A. veronii* genomes, suggesting that this strain has been exposed to different environments and acquired unique phage elements. Phage vB_AbaM_ME3 was previously isolated from wastewater effluent using the propagating host *Acinetobacter baumannii* DSM 30007 (Kropinski et al., 2009). *A. baumannii* is a known nosocomial pathogen that causes pneumonia, urinary tract infection, and septicemia (Buttimer et al., 2016). Phage vB_AbaM_ME3 of *A. baumannii* has a size of 234,900 bp and 326 ORFs (Buttimer et al., 2016). The Myovirus-type phage RSA1 is relatively small (39 to 40 kb) and has lytic activity. It has restricted host range, mainly *Ralstonia solanacearum*, a soil-borne species pathogenic to many important crops (Yamada et al., 2007; Addy et al., 2019).

Interestingly, the MS-17-88 genome has four prophages sharing structural similarities with temperate Aeromo_phiO18P elements found in *Aeromonas media* isolated from a pond in Germany (Beilstein and Dreiseikelmann, 2008). The phiO18P phage type belongs to the *Myoviridae* phage family and consists of 33 kb. The phiO18P phage elements typically have 46 ORFs encoding proteins responsible for integration and regulation, replication, packaging, head and tail, and lysis (Beilstein and Dreiseikelmann, 2008). Unlike other prophage elements, Aeromo_phiO18P does not have a lytic phase; it replicates lysogenically by integrating its genome into the bacterial chromosome (Vincent et al., 2017). In some instances, the phage-encoded genes are advantageous to the host bacteria (Dziewit and Radlinska, 2016). Aeromo_phiO18P shows significant similarity to the P2 phage family in *Aeromonas salmonicida* and *Vibrio cholerae* K139 genomes (Beilstein and Dreiseikelmann, 2008).

**Figure 5:**
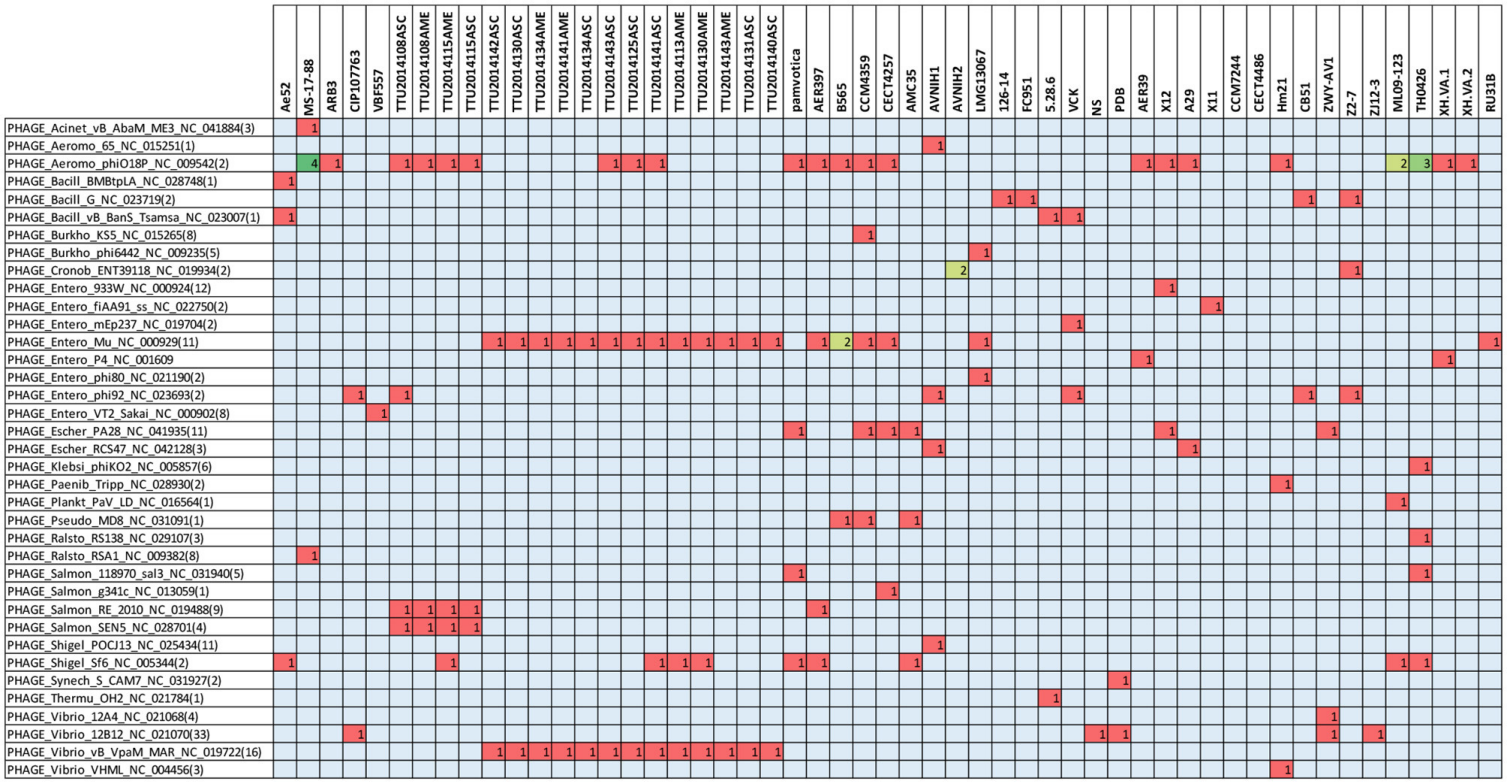
Type of prophage elements present in the *A. veronii* genomes. Red color represents presence of the gene.

The present study documented 40 different types of phage elements across the 53 *A. veronii* genomes (Figure 5). Among these 40 phage elements, phage type “Aeromo_phiO18P” is the most abundant type in all the evaluated *A. veronii* genomes as well as the most abundant in strain MS-17-88. Strains CCM 4359 and XH.VA.1 carry five different phage elements. Incomplete Staphy_SPbeta_like phage element was detected in two strains (MS-17-88 and AVNIH1). In our initial analysis of the MS-17-88 genome using PHASTER, we identified that the strain carries the *floR* gene inside incomplete phage element PHAGE_Staphy_SPbeta_like_NC_029119 (genome position 2631302-2644102), but later analysis with PHASTER showed that this may not be a true phage element. Further investigation of this region is warranted to determine whether a phage element mediated dissemination of the *floR* resistance gene (Garriss et al., 2009).

### Comparative Analysis of Antibiotic Resistance Determinants

**Figure 6:**
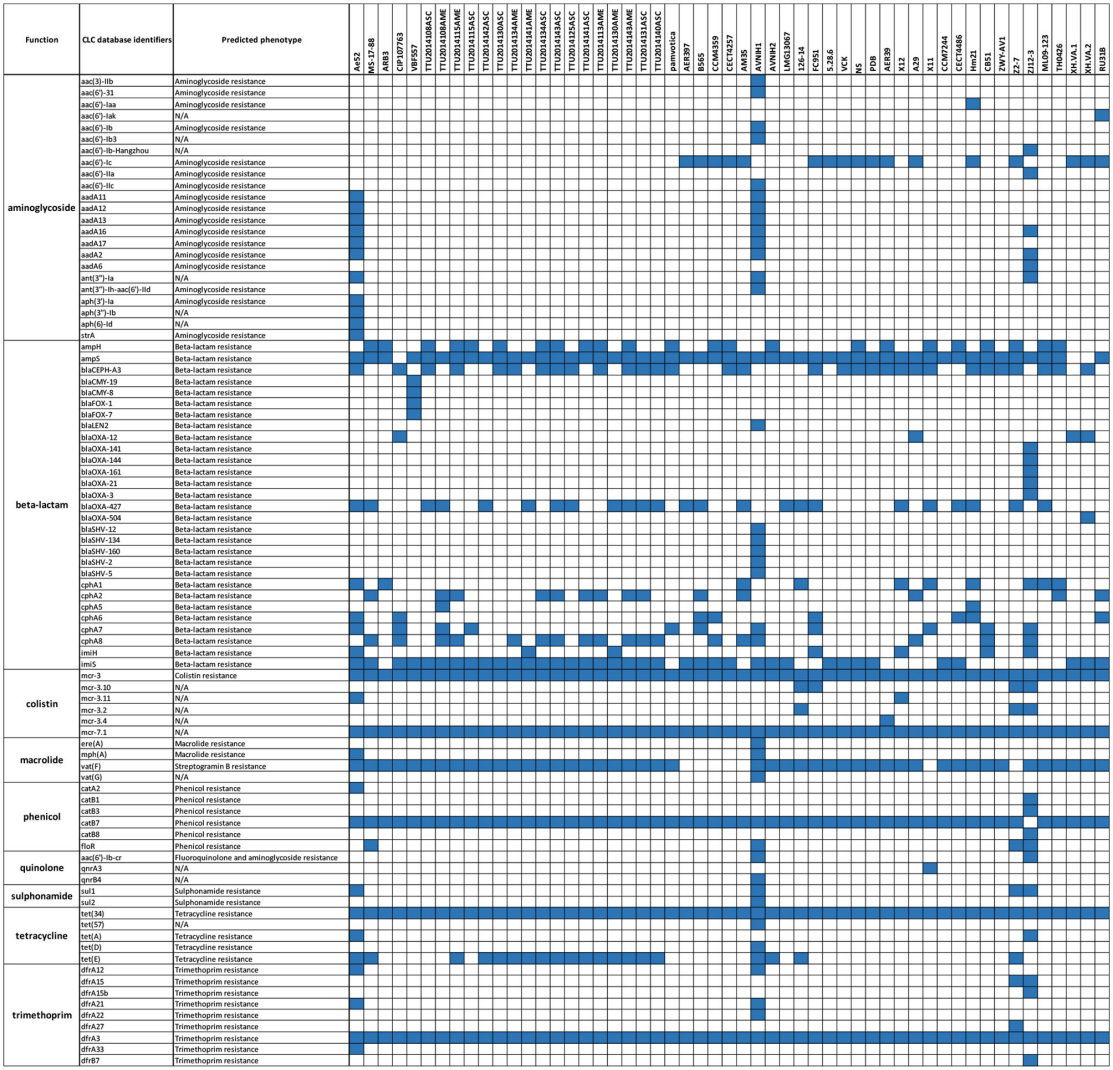
AMR genes distribution across the 53 *A. veronii* genomes. Blue color represents presence of the gene.

A comparative analysis of 87 AMR determinants and components was conducted to determine their distribution among the 53 *A. veronii* strains. *A. veronii* MS-17-88 shared a common AMR gene composition with the other *A. veronii* genomes. Figure 6 shows the distribution of antibiotic resistance genes in each strain. All the *A. veronii* genomes carry tetracycline [*tet(34)* and *tet(E)*], and trimethoprim (*dfrA3*) resistance genes. Oxytetracycline, tetracycline, and trimethoprim/sulfamethoxazole have been extensively used in human clinical, veterinary, and agricultural sectors for decades. The linkage between AM use and resistance has been demonstrated for other bacteria in aquaculture ecosystems and other animal husbandry facilities (Verner-Jeffreys et al., 2009; Lagana et al., 2011; Tamminen et al., 2011). In addition to *tet(34), tet(E)*, and *dfrA3, A. veronii* MS-17-88 genome carries colistin resistance genes (*mcr-7.1* and *mcr-3*). There have been an increasing number of reports on the identification of *mcr* genes in many bacterial species globally (Stoesser et al., 2016; Elbediwi et al., 2019). A recent study reported that *mcr-3* variants are more common in Aeromonas than in other bacterial species but aeromonads do not inherently carry the *mcr-3* gene (Shen et al., 2018). However, it is speculated that Aeromonas isolates from the aquatic environment may be the major reservoir for the dissemination of *mcr-3* genes to other bacteria (Ling et al., 2017; Eichhorn et al., 2018). Furthermore*, A. veronii* MS-17-88 genome harbors a macrolide resistance gene *vat(F)* that encodes an acetyltransferase that acetylates class A streptogramins (Seoane and García Lobo, 2000). Six genes encoding resistance to β-lactamases were identified in *A. veronii* MS-17-88 genome including *ampH* and blaOXA-427 belong to class D beta-lactamase, ampS encoding a class 2d penicillinase and hydrolyzing mainly penicillins (Walsh et al., 1995), and *cphA2, cphA8*, and *imiS* encoding a class 3 metallo-β-lactamase and active mainly against carbapenems (Walsh et al., 1998).

Among the 53 *A. veronii* strains, AVNIH1 strain had resistance genes to almost all the antibiotic classes. This clinical strain was isolated from human stool, and the genome exhibits clear evidence of horizontal gene transfer (Hughes et al., 2016). In our analyses, we did not observe any pattern of antimicrobial resistance in specific bacterial host or sources. However, there was one exception: the highest number of AMR elements were observed in two human isolates (strain AVNIH from U.S.A; (Hughes et al., 2016) and ZJ12-3 from China; Shen et al., 2018), and one isolate from septicemic goldfish (strain Ae52 from Sri-Lanka; Jagoda et al., 2017). The use of antimicrobial agents in human medicine may be associated with these nosocomial trends (Hughes et al., 2016).

In regard to sulphonamide resistance*, sul1* was detected in four strains (Ae52, ANIH1, Z2-7, and ZJ12-3) representing 7.5% of the *A. veronii* strains, and *sul2* was present in one strain (Z2-7) representing 1.8%. This suggests that *sul1* is the most frequent gene encoding sulfonamides resistance in *A. veronii*. Nearly all strains (100%) carried *tet(34)*, and a significant proportion of strains carried *tet(E)* (20 strains; 37%). In contrast*, tet(57)* and *tet(D)* were detected in only one strain (ANIH1) (1.8%), and *tet(A)* was detected in two strains (Ae52 and ZJ12-3) (3.8%). *floR* gene conferring resistance to florfenicol and chloramphenicol was present in only four strains (MS-17-88, AVNIH1, Z2-7, and ZJ12-3) (7.5%). The prevalence of beta-lactam resistance genes in the 53 *A. veronii* genomes was as follows: *ampH* was detected in 41.5% of the strains, *ampS* was detected in 92.4% of the strains, *imiH* was detected in 13.2% of the strains, *imiS* was detected in 71.7% of the strains, blaCEPH-A3 was detected in 56.6% of the strains, blaOXA-427 was detected in 45.3% of the strains, *cphA1* was detected in 17% of the strains, *cphA2* was detected in 26.41% of the strains, *cphA6* was detected in 13.2% of the strains, *cphA7* was detected in 20.7% of the strains, and *cphA8* was detected in 44% of the strains. Of the aminoglycoside resistance genes, *aac(6*′*)-Ic* was the most prevalent (32%). Interestingly, *A*. *veronii* AVNIH1 and Ae52 strains carried multiple aminoglycoside-resistance genes. Several studies reported that the majority of *Aeromonas* species exhibit only a single aminoglycoside modifying gene (Dahanayake et al., 2019). However, *Pseudomonas aeruginosa* isolated from hospitals from Iran was reported to carry up to four aminoglycoside resistance genes (Perez-Vazquez et al., 2009).

In conclusion, we used genome sequencing to investigate genetic variation and AMR gene distribution in 53 *A. veronii* genomes. We found significant genetic differences and a high degree of genomic plasticity in the evaluated *A. veronii* genomes. Overall, the AMR gene frequency against sulfamethoxazole and florfenicol is low, while AMR genes against tetracycline are very high. Among tetracycline-resistant isolates, *tet(34)* and *tet(E)* were the most frequent AMR genes. Taken together, our results show that AMR genes are common and are distributed among *A. veronii* genomes; however, the frequency of most AMR genes in individual strains is still low. Identified phage elements may be useful for future development of an efficient and effective bio-treatment method to control bacterial diseases in aquaculture. The knowledge generated from this study can benefit our understanding of *A. veronii* evolution and provide insight into how *A. veronii* isolates are intrinsically resistant to multiple antimicrobials. In addition, *A. veronii* species are important considerations as potential sources for resistance determinants in the environment. Therefore, it is important to continue surveillance of resistance and genetic mechanisms of resistance in this species.

## Data Availability Statement

The datasets presented in this study can be found in online repositories. The names of the repository/repositories and accession number(s) can be found in the article.

## Author Contributions

HT, ML, and HA designed and conceived the analysis and experiments. HT, MA, C-YH, AT, JB, and HA performed the experiments and analyzed the data. HT, ML, and HA wrote the manuscript. All authors read and approved the final manuscript.

## Conflict of Interest

The authors declare that the research was conducted in the absence of any commercial or financial relationships that could be construed as a potential conflict of interest.
